# Texas State Medical Association, Nineteenth Annual Meeting; Synopsis of Proceedings

**Published:** 1887-05

**Authors:** 


### SOCIETY NOTES.

### TEXAS STATE MEDICAL ASSOCIATION.

**Nineteenth Annual Meeting, Austin, Texas, June 26, 27, 28, and 29, '87.**

### SYNOPSIS OF PROCEEDINGS.

MORNING SESSION, FIRST DAY, TUESDAY, 26.

The meeting was called to order by Dr. J. W. McLaughlin, Chairman of committee of arrangements, at 10-30 a. m., Tuesday, April 26. Rev. T. B. Lee, Rector of St. David's Episcopal Church, delivered an eloquent and impressive prayer, in verse.

Dr. Daniel, chairman of Committee of Reception, in a brief address, extended a welcome on behalf of Travis County Medical Association, and introduced Hon. J. W. Robertson, Mayor of Austin, who made an appropriate address of welcome. He was followed by Mr. A. H. Graham, who, being introduced, delivered an address also, appropriate, very eloquent and interesting. We regret that we cannot reproduce these addresses here, for want of space; they will appear in the transactions.

The President, Dr. T. H. Nott, of Goliad. was escorted to the chair by Drs. Morris and Wooten, of the reception committee, and delivered his opening address, replying in appropriate terms to the eloquent and fervent addresses of welcome.

The session was opened by calling the roll of members, and of county associations in affiliation. There were present and answering to their names, 93 members, and representatives from seven auxiliary societies, though there were probably double that number in the room, many being, as yet, not connected with the association, but delegates from counties and county societies.

The President delivered his annual message, recommending a new constitution and by-laws—the one at present governing the association being inadequate to its wants; the appointing of a board of censors of five, to be added to the permanent committees, and their duties defined, they to take cognizance of and adjust all matters of a "personal character, and minor grievances;" that "the stigma of professional disgrace should rest upon any who allow their names to be advertised as the endorser of patent medicines," etc; that the reports of chairmen of sections, if over ten pages, be referred to publishing committee without being read, etc.; that advertisements be excluded hereafter from the published transactions of the association, etc.

The reading of the the minutes of Dallas meeting was, on motion, dispensed with, and adopted as printed.

On motion of Dr. H. L. Parsons the message of the President was referred to special committee of five, with instructions that that portion relating to revision of constitution and by-laws be referred to the committee appointed for same purpose last year at Dallas. Committee: Drs. D. F. Stuart, M. D. Knox, H. K. Leake, J. D. Osborn, H. C. Ghent.

The judicial council was called, and the following members were present: Drs. H. M. Oliver, Frank Allen, M. L. York, H. H. Thorpe, T. J. Tyner, M. L. Haggard, J. R. Johnson. The following were added: Drs. J. W. Garnett, H. H. Darr, O. L. Williams, J. L. Irion, and G. W. Kerr. The judicial council immediately went into session.

Communications were read from a large number of distinguished physicians throughout the U. S., and from absent members, expressing regrets, and sending greeting. They were ordered to be filed with the archives of the association.

The superintendents extended invitations to the delegates to visit the several State Institutions, the Blind Institute, Lunatic Asylum, Deaf and Dumb Asylum, the new capitol, etc; accepted with thanks.

Governor and Mrs. Ross invited the delegates and their ladies to a reception at the Mansion on Wednesday evening, and Dr. J. W. McLaughlin also tendered them a reception. A vote of thanks was recorded.

Dr. J. F. Y. Paine, chairman of Section on Practice, read a valuable paper on Fevers—his report as chairman. On motion it was referred to publishing committee with instructions to publish it in the transactions. Adjourned.

AFTERNOON SESSION, APRIL 26.

Section on Practice called: Dr. J. F. Y. Paine, Chairman, presiding.

The following papers were then presented:

First—Some hints on the action of Antipyrine, by Dr. O. L. Williams, of Chappel Hill. Referred to the publishing committee.

Second—Mental Alienation, a Sequel of Intermittent Fever, by Dr. E. Goldman, of Galveston. Referred to publishing committee.

Third—Babies and Their Troubles, by Dr. C. L. Gwyn, of Galveston. Referred to publishing committee.

Fourth—Spinal Irritation, by Dr. J. W. Carhart, of Lampasas. This paper evoked a spirited and interesting discussion, in which Drs. Williams, Dudley and Talley participated. The paper was referred to the publishing committee to be published, and a vote of thanks was tendered to Dr. Carhart.

Fifth—Typho-Malarial Fever, by Dr. J. E. Ward, of Waxahachie. Referred to publishing committee.

Sixth—Pneumonia, by Dr. J. C. Milner, of Comanche. Referred to publishing committee.

Seventh—A case of Hæmatemesis of Obscure Causation, by Dr. C. F. Payne, of Comanche. Referred to publishing committee.

On motion of Dr. Daniel the paper of Drs. Gwyn and Goldman were also ordered to be published.

Dr. S. T. Lowry, of San Antonio, read a paper on "Dermatitis produced by the Administration of Quinine."

This case presented some remarkable features, and has become quite notorious. It is that of a lady—the wife of a railroad engineer, who had, on taking quinine, suffered with a rash, followed by desquamation. It was in Dr. Wilkinson's hands, and the case is reported in one of the Texas journals. Dr. Wilkinson, it seems, disregarded the lady's injunction that she could not take quinine. When she fell into Dr. Paine's hands he did the same, and the administration of the drug produced the same results; afterwards Dr. Lowry had a similar experience, and related the case. The suffering was intense, and the desquamation was complete, the epidermis coming off in large flakes, casts of the part shedding.

Dr. Cummings and Dr. Hewson and Dr. West related cases of urticaria following the use of quinine; sulphate cinchonidia did not produce it, while in the above cases cinchonidia and quinine had the same effect. Dr. Lowry's paper was referred to the publishiug committee.

Drs. Bowers, Coleman and Lancaster jointly reported a case of "Typhlitis and Perityphlitis" caused by the ingestion of large quantities of acorns, in a child of 12 years—a most remarkable case. Referred.

These papers were discussed at length, and elicited much interest. As we can give only a synopsis of the four days proceedings, the discussion is necessarily omitted—much to our regret. It will appear, however, in the transactions.

The Judicial Council made the following report:

"Motion unanimously carried to recommend that Dr. W. G. Hardin, of Terrell, on charges and specifications preferred, be expelled."

"Motion unanimously carried to recommend that Dr. F. W. Kaiser, of Flatonia, on charges and specifications preferred, be expelled."

"Motion made and carried to receive Dallas County Medical Association in affiliation, with the admonition that they take better care of their society at home." Delegates, Drs. E. L. Thompson, J. A. Ewing, S. D. Thurston and H. K. Leake.

This gave rise to some spirited discussion, and reasons were demanded for the reprimand to their society, by the Dallas delegates. Dr. Chilton, of the Dallas County Society explained that he had gone before the council and had given information in the interest of the society; that there were certain irregularities practiced by certain members, not members of the State Association, and which they had not been able to correct, and had asked for an admonition in the hope of causing said members to conform to the code. He instanced "Dr. So and So," of his society, whose card and sign were not in accordance with the code of ethics. This statement appeared satisfactory, but that part of the Council's report to which objection was made, was, on motion, referred back to the Council.

The proposal to expel the above named members also gave rise to discussion and objection. Drs. Osborn and Douglas objected to voting on the proposition to expel a member without knowing why, and a call was made for the evidence. This was produced, and by request, was read from the secretary's desk. The charges in Dr. Hardin's case were violations of the code of ethics, and the specifications were—manufacting and selling in copartnership with a well known irregular, a patent medicine, under the name of the "Red Line Medicine Company," and for advertising in the newspapers. The charge vs. Dr. Kaiser, preferred by Dr. Paulus, a member of the Association and of the Judicial Council, and by Dr. Britton, also a member of Association, recited that Dr. Kaiser was in copartnership with a well known quack and that his friends had remonstrated with him, in vain, pointing out the consequences, which he disregarded.

On motion, the report of the council was adopted. Adjourned.

WEDNESDAY, APRIL 27—MORNING SESSION.

The meeting was called to order by the President at 10 a. m. There was a great increase in the attendance, and the clerks and treasurer were kept busy with the register.

Communications were read from Dr. Hunter McGuire, of Richmond, Virginia. and Dr. T. C. Osborne, (an honorary member) of Cleburne; also, Dr. S. H. Stout, of Cisco, regretting their inability to be present; also, from the Galveston Medical society, inviting the association to hold its next session in that city. The mayor of Corpus Christi also sent a cordial invitation to meet there next year. Invitations to visit the new capitol, the John B. Hood Camp, and the University, were likewise received.

Dr. J. H. Sears asked to be excused from further services on the judicial council; Dr. G. W. Kerr, of Waelder, was appointed in his stead.

The annual report of the secretary, Dr. F. F. Daniel, was read, as follows:

### SECRETARY'S REPORT.

### NINETEENTH ANNUAL CONVENTION TEXAS S. MEDICAL CONVENTION.

AUSTIN, TEXAS, April 26, 1887.

DR. T. H. NOTT,
President Tex. S. Med. Association.

SIR: As Secretary, *ad interim*, by your appointment to the vacancy occasioned by the death of Dr. W. J. Burt, I have the honor to submit the following report:

AUSTIN, TEXAS, April 26, 1878.

F. E. DANIEL, M. D., SECRETARY AD INTERIM, IN ACCOUNT WITH THE TEXAS STATE MEDICAL ASSOCIATION.

| | | |
|---|---|---|
| 1886–7 Dr. | | |
| April, 1887, To Cash, Dues from Members: | | |
| August, '86, To Cash, Dr. E. L. Sessions......$ | 5 00 | |
| August, '86, To Cash, Dr. R. M. Swearingen .. | 5 00 | |
| Sept. 17, '86, To Cash, Dr. W. W. Walker.... | 5 00 | |
| Sept. 24, '86, To Cash, Dr. F. P. McLaughlin. | 5 00 | |
| Oct. 6, '86, To Cash, Dr. A. D. Paulus........ | 5 00 | |
| Nov. 7, '86, To Cash, Dr. J. C. Jones........ | 5 00 | |
| Jan. 14, '87, To Cash, Dr. J. M. Litten........ | 10 00 | |
| March 22, '87, To Cash, Dr. T. S. Burke...... | 5 00 | |
| Feb., '87, To Cash, Dr. T. H. Nott........... | 10 00 | |
| April 7, '87, To Cash, Dr. T. D. Wooten...... | 10 00 | |
| April 7, '87, To Cash, Dr. W. A. Morris...... | 10 00 | |
| April 7, '87, To Cash, Dr. C. R. Hargrove.... | 5 00 | |
| April 10, '87, To Cash, Dr. F. Paschall, Mexico | 10 00 | |
| April 13, '87, To Cash, Dr. E. N. Wright, I. T. | 5 00 | |
| April 13, '87, To Cash, Dr. J. L. Lankford, I. T. | 5 00— | $ 100 00 |
| Nov. 1, '86. To Cash, Draft on Treasurer..... | 942 00 | |
| Dec. 23, '86, To Cash, Draft on Treasurer.... | 25 00 | |
| To Cash, Sale of Transactions.............. | 6 20 | |
| March, '87, To Cash from Publishing Com'tee | 255 00— | 1228 20 |
| | | $1328 20 |

Cr.

| | | |
|---|---|---|
| Nov. 30, '86, Paid Warner & Draughon, v. 2... | $1089 65 | |
| March, '87, Paid John Southgate, v. 3........ | 26 50 | |
| Nov., '86, Paid Exp. on 428 vols. at 21c } v. 5. | 89 89 | |
| Nov., '86, Paid Express on 6 vols. (1 pkg) } v. 5. | 40 | |
| Nov., '86, Paid Ex., Pacific, 86 vols. at 21c, v. 4 | 18 06 | |
| Aug., '86, Paid Postage, 200 circulars........ | 2 00 | |
| Feb. or March, Paid Postage Pres't Circular.. | 10 00 | |
| Nov., '86, Paid for Stamps, 76 vols. at 24c, v. 6 | 18 24 | |
| April 8 and 13, '87, Paid for Stamps, 428 Report Surgery, v. 10...................... | 29 95 | |
| Nov. 15, '86, Paid Warner & Draughon, circulars and letter heads, etc., v. 1........... | 8 50 | |
| Nov. '86, Paid Exchange on $942 at ¼ pr. ct. | 2 35 | |
| April, '87, Paid return charges on 7 vols., v. 9. | 1 89 | |
| March, '87, Paid for folding and st'ping circulars, v. 7.......................... | 2 00 | |
| April, '87, Paid for stamps and postals for office, 1 year........................... | 7 00 | |
| April, 1887, Paid Express on Books Dr. Cupples, v. 8 .......................... | 35 | |
| April, '87, Paid Cartage on Transactions 3 times, | 75 | |
| April, '87, Balance........................ | 20 59 | |
| | $1328 20 | $1328 20 |
| April 26, '87, To Balance.................. | | $20. 59 |
| April 18. Received since, R. A. Taylor, Millwood, | 5 | |
| April 20. " " J. T. Harrington, El. P., | 10 | |
| April 25. Received since, Dr. H. C. Ghent,.... | 5 | |
| April 25. " " A. W. Fly, Galv.... | 5 | |
| " " W. M. Powell, Albany, | 5 | 30 00 |
| By cash h'nd. Treas. with Dr. Harrington's Ap. | 10 | |
| By Balance handed Treasurer,.............. | 40 59 | $50 59 |

E. & O. E. Austin, Texas, April 26, 1887.

With the exception of publishing and delivering the Transactions for 1886, it will be observed the expenses of the office have been unusually light. The correspondence has not been extensive, amounting to only a few hundred letters and postal cards. The only circulars issued from the office were those of the President to delinquents, in August, (200) and the annual announcement and call for meeting, which were issued in February or March.

The delay in distributing the Report on Surgery was occasioned by want of funds, and the last were mailed on the 13th inst. About 450 copies were mailed from the Secretary's office, and some 25 delivered in Austin. The Transactions were promptly mailed and sent by express, upon delivery, and a larger number has been distributed than any previons year.

Since last meeting it is my duty to report the death of seven honored and useful members—an unusually large mortality, and tho' I am aware the committee on necrology will do full justice to their memories, I cannot refrain from at least a passing mention:

Dr. Joseph Willis, of Waco, died July 6.
" W. J. Burt, of Austin, died July 10.
" H. W. Moore, of Fort Worth, died September 24.
" Albert Welch, of Ferris, died Semtember 22.
" J. T. Meek, of Ennis, died November 11.
" T. M. Stone, of Jasper, died Sep. 14.
" James Haley, of Moffatt, died February 11, 1887.

The actual number of members in good standing at present borne upon the roll is 417. Of this number three have removed from the State, to-wit: Dr. W. H. Wilkes, late of Waco, Dr. B. M. Brown, and Dr. Wm. Penny, of Galveston. Two have tendered their resignations herewith presented, to-wit: Drs. B. M. Brown and D. B. McMillan. Of those dropped from the roll for non-payment I find no record by my predecessor. Two who had formerly been dropped have been re-instated under the resolution adopted last year at Dallas: Dr. W. A. Morris, of Austin, and Dr. Frank Paschal, of Chihuahua, Mexico.

The roll of members is very imperfect, and incomplete. Until the present time names of members, long since dead, or removed, are carried on the roll ; while quite a number have changed their place of residence, without giving notice to the Secretary's office ; and tho' repeated and urgent calls have been made, both by circular and through the medium of the Medical Journals for data to enable the Secretary to complete the record, as to place and date of graduation, when joined the Association, etc., it has been in vain ; only very few have responded.

The failure to report change of residence, entails considerable trouble and some expense,—in as much as valuable Transactions sent by express or mail, prepaid, are returned with the remark, "no such person resides here," and return charges are also paid by the Secretary. Seven instances of the kind occured with the last volume of Transactions,—and one was in the case of a member who only joined at Dallas.

Of County and Town Associations in affiliation with the State Association there are only seventeen borne on the roll—tho' I believe, there were one or two represented at Dallas, whose names do not appear : and a singular dereliction on the part of somebody is —the name of not a single Secretary is given. In this connection, Mr. President, I hope I will be pardoned for calling attention to the requirement of our by-laws, which provides for annual reports from these auxiliary societies as to the membership, the work done, etc. In not a single instance has a report been made this year.

An inventory of the books in possession of the Secretary reveal the nucleus of what can be made by proper care and attention—a *library:*

There are 59 volumes of State Society Proceedings in pamphlet form, and 32 volumes in cloth, of Transactions of State Boards of Health and State Medical Associations.

Of our own Transactions I do not find on hand a single volume later than 1883, (1 volume only, of 1883, in paper), and of the year 1884, there are in paper covers, 138; and in cloth covers, 1; of the year 1885, there are in cloth covers, 108; of the year 1886, there are in cloth covers. 74; total 321; including the seven or eight returned volumes; and 125 copies of the Committee's Report on Surgery. Thus, in all, there are some 537 books on hand, the property of the Association. These have been carefully arranged on shelves, and I would suggest that they are worth the cost of a book-case, or other means for their proper care. It would seem that no better use could be made of the back volumes of Transactions than to give them to the new members.

Respectfully submitted,

F. E. Daniel, Secretary.

Dr. Terhune moved that the Secretary's report be adopted. Dr. J. B. Robertson moved that it be referred to a special committee of three. Carried. Drs. R. T. Knox, F. M. Pitts and A. A. Terhune, were appointed. They carefully examined the report and finding it correct in every particular, so reported.

The report of the Treasurer Dr. J. Larendon, was read as follows:

### TREASURER'S REPORT.

Houston, Texas, April 20, 1887.

Dr. J. Larendon in account with Texas State Medical Association.

RECEIPTS.

| | |
|---|---|
| 1886-7. | |
| April 21, Balance on hand, last report.......$ | 45 52 |
| April 20, Annual dues collected............ | 1,894 25 |
| | $1,939 77 |
| April 20, '87, To Cash on hand............. | 20 07 |

DISBURSEMENTS.

| | |
|---|---|
| 1886-7. | |
| April 29, Cash paid Collective Investigation Committee, per order..................$ | 42 75 |
| April 29, Cash paid for Stationary.......... | 5 50 |

| | |
|---|---|
| April 30, Cash paid Secretary salary........ | 200 00 |
| Cash paid Publishing Committee for work of 1885............................ | 200 00 |
| Cash paid Treasurer........................ | 100 00 |
| Cash paid Postage Stamps.................. | 3 00 |
| June 18, Cash paid Dr. Geo. Cupples, for work on Surgery, $294 00; P. O. M. O. fee, $1 35 ............................ | 295 35 |
| August 16, Cash paid Dr. J. R. Briggs, for Prize Essay.......................... | 100 00 |
| Exchange on above ...................... | 45 |
| Nov. 1, Cash paid F. E. Daniel, Chairman Publishing Committee................. | 942 00 |
| Dec. 23, Cash paid F. E. Daniel, Chairman Publishing Committee................. | 25 00 |
| P. O. M. O. on above..................... | 15 |
| April 19, '87 Cash paid A. C. Gray, printing blank receipts, 1000...................... | 5 50 |
| Balance Cash on hand..................... | 20 07 |
| | $1,939 77 |

Respectfully submitted,

E. and O. E. J. Larendon, Treasurer.

On motion, the report was referred to same committee. On examination it was found correct, and so reported. Dr. Pitts complimented Dr. Larenden on the report, and moved that he be paid $100 for his services. Amended by Dr. J. B. Robertson, by adding $10 for postage. Carried.

The following report of the Publishing Committee was submitted by Dr. F. E. Daniel, chairman:

REPORT OF PUBLISHING COMMITTEE.

*Dr. T. H. Nott, President Texas State Medical Association:*

Sir.—Your Committee on Publication beg leave to submit the following report:

The volume of Transactions for 1886, eighteenth Annual session, is herewith presented.

Seven hundred volumes, of seven hundred pages each, were issued, and a copy was sent to every member of the Association whose name appears on the roll; to every county medical association in affiliation, whose secretary's address is known; to most of the secretaries of State Medical Associations, and State Boards of Health; to the officers of other medical and sanitary associations, the American Medical Association and the American Public Health Association, U. S. Government medical officers; to many medical colleges, and to the leading daily newspapers in the Southern State, and to nearly all of the leading medical journals; also to the advertisers—in all, some six hundred and thirty volumes; a larger number than have ever heretofore been distributed from the Secretary's office.

The printing was done by Messrs. Warner & Draughon, and the binding by Mr. John Southgate, all of Austin,—according to contract with the first named—at a net cost of $1089.65; or $1.55 per volume.

Of this amount, $861.15 in the Treasury was available; the balance being required to prepay postage and express charges. The postage was 24 cents per volume, weighing 48 ounces (3 lbs.), and the express charges (special rate to committee) was 21 cents per volume. The balance of the amount was advanced by the committee, as per statement below:

| | | |
|---|---|---|
| Dr.—Voucher No. 1, Warner & Draughon bill, printing 700 volumes | | $1089 65 |
| Voucher No. 2, John Southgate's bill for wrapping, papers, printing same, labor, twine, pasting in 1400 slips, etc. | | 26 50 |
| Total | | $1116 15 |
| Cr.—Voucher 1, By cash from Secretary | $861 15 | |
| Voucher 2, By cash by Publishing Committee | 255 00 | 1116 15 |
| Balance due Publishing Committee | | $ 255 00 |

The manuscript of the Report on Surgery was withdrawn from the hands of the Publishing Committee by the chairman of special committee on said report, who made special contract with Messrs. Warner & Draughon; he also claimed the privilege of reading the proofs, and of supervising the work. The Publishing Committee are, therefore, entitled to none of the credit for the excellent publication.

The amount of manuscript placed in the hands of the Publishing Committee was immense—far exceeding any previous year; and the labor of classifying and arranging it, revising, and correcting clerical and other errors, and putting it in shape for the press, involved no small amount of time, patience and labor; all of which was shared by the lamented Burt up to the time of his death; and the subsequent reading and revision of the large amount of proof was a labor of many weeks. The work speaks for itself. Of course it is not perfect; but we believe it is as nearly free from typographical errors, and other blemishes, as most works of the kind, and as three careful proof-revisions, in very trying weather, could well make it.

It is a source of much gratification to your Committee, and, we hope, of pardonable pride, to observe the very general favor with which their labors have been, everywhere, received; the palm for excellence,—not alone for the mechanical part of the volume, but for the high scientific value of its contents, has been, by the medical press of the country, with one accord, awarded to the Transactions of the Texas State Medical Association!

Believing that the members will share that feeling with the committee, and that it will stimulate them to renewed efforts, your committee hope to be pardoned the seeming vanity which prompts them to submit herewith a few brief extracts from the many kind and complimentary notices of the work, which have appeared in the medical journals of the country. The journals of nearly every State have spoken kindly of our humble but earnest labors. and in praise of the enterprise,—and the scientific ability of the Texas medical profession. [Comments of the press are omitted for want of space.]

Thus, the journals from nearly every State in the Union have been pleased to compliment the profession of Texas and their scientific work as exemplified in their published Transactions.

Respectfully submitted.

F. E. DANIEL, Ch'mn,
R. M. SWEARINGEN,
J. W. McLAUGHLIN.

AUSTIN, TEXAS, April, 1887.

Dr. Daniel also made a verbal report, stating that he and Dr. Burt had received each $146 from the advertisers, and explained the difficulties attending the publication of the volume.

The report was adopted, and, on motion of Dr. G. W. Christian, a vote of thanks to Dr. Daniel was recorded; and, on motion of Dr. Swearingen, $100 was voted from the treasury as additional compensation for his time and labor in editing and publishing the volume of Transactions.

Dr. S. D. Thurston, of the Committee on Prize Essays, rose to a point of personal privilege, and asked to be permitted to make a statement in regard to the matter and manner of making the award of the prize last year. He said that the published article of Dr. Wallace, one of the committee, in DANIEL'S TEXAS MEDICAL JOURNAL, reflected unjustly on the committee, and called for a reply.

On motion of Dr. A. P. Brown, the rules were suspended, and Dr. Thurston read an able and elaborate article defending the action of the committee, and answering Dr. Wallace on all points, and describing the manner in which the award was made.

On motion of Dr. Becton—after several motions had been made and amendments offered—the following resolution, which embodied the main idea, was unanimously adopted:

*Resolved*, That the personal explanation of the Committee on Prize Essays be accepted as entirely satisfactory to the Association, and that their manner of awarding the prize be, and is hereby, in all things, approved. E. P. BECTON.

Dr. Geo. Cupples, Chairman of the Committee on Collection of Surgical Cases, made the following

REPORT ON SURGICAL CASES.

*T. H. Nott, M. D., President Texas State Medical Association;*

SIR.—Your Special Committee on Surgery have the honor to submit herewith their annual report for 1886, comprising 1046 operations, which, added to 4293 operations included in the report presented at the last meeting, make an aggregate of 5339 operations presented.

It may be interesting to note here, as an evidence of the correctness and accuracy of the individual reports, that the mortality in the first report furnishes a ratio of 8.15 per cent, whilst the present report furnishes a death rate of 7.93 per cent.

The present report comprises many operations not heretofore included and not strictly belonging to this year, and comprises a large number of the less dangerous operations.

In preparing this report the same method was followed as on former occasions; 3000 blank forms were distributed to physicians throughout the State, and a very large correspondence was entered into in connection therewith; seventy-four reports were received from sixty-six surgeons, which are embodled in the document now presented for your acceptance.

Some explanation is due to the Association of the delay which occurred in the printing and distribution of the report of 1886. Certain difficulties arose regarding the printing of the report as ordered by a vote of the Association. The difficulties were met and surmounted by the action of a few members, who provided for the printing of 1000 copies. In doing this, however, they were hampered by a contract entered into by the publishing committee for the printing of the entire Transactions, which caused great delay and many typographical errors, due to the unfaithful execution of the contract by the printers.

Notwithstanding these drawbacks, 400 copies were forwarded in January by the committee to the individual reporters and to many eminent men in the profession in the United States and in Europe, from many of whom have been received the most gratifying expressions of approval and commendation of the scope and value of the work.

As a result, the surgeons of Texas are to-day regarded and treated as no unworthy members of the great republic of work.

It is to be hoped that the work will be continued, and, to use the words of a reviewer of the report, that a lesson will be learned by the other State Medical Associations from the action of that of Texas.

From the nature of the undertaking, it became necessarily the work of one member of the committee, who is alone responsible for errors contained therein, and who gratefully takes occasion to acknowledge the intelligent and indefatigable assistance rendered by his friend, Dr. E. H. Tyler, of San Antonio, without whose aid this arduous and laborious undertaking could never have been accomplished.

All of which is respectfully submitted.

[Note.—Four hundred and fifty copies were subsequently mailed to members by the Secretary, and the balance sent to the chairman of the committee.—Ed.]

On motion, the report was adopted, and a vote of thanks was tendered to Dr. Cupples.

Dr. Robertson moved that Dr. Cupples be reimbursed for the expense of publishing the extended report on surgery. Carried.

Dr. Cupples begged to decline the reimbursement.

Dr. H. L. Taylor, delegate to the Pharmaceutical Association, asked for further time in which to make his report. Granted.

Dr. Kilpatrick, chairman of the Committee on Necrology, not being ready to report, was given further time.

Vice-President Parsons, who was occupying the chair, at this point suspended the regular business and introduced Dr. W. H. Saunders, Professor in the Alabama Medical College, and a representative from the Alabama Medical Association to the Texas Medical Association. Upon being presented, he made a brief address, which was full of fraternal greeting and cordial fellowship. His remarks were fitting and appropriate, and were received by the Association with generous rounds of applause.

Dr. J. B. Robertson, addressing Professor Saunders, bade him welcome to the bosom of the Association, and thanked him for his coming, at the same time complimenting in high terms the sister Association which was so ably represented here in his person. Dr. Becton also made a highly complimentary speech, eulogizing the Association from which Dr. Saunders was a representative.

Dr. H. W. Dudley offered a resolution providing for the severance of venereal diseases from the surgery section, and the creation of a section upon those diseases. A motion to refer to the Committee on Constitution and By-Laws was superseded by a motion to table, which prevailed.

Dr. Cummings offered a resolution that all applications for membership, and all charges and specifications against members, be submitted in writing to the Association, and read from the Secretary's desk before reference to committees. The resolution was, on motion, referred to the Committee on Constitution and By-Laws.

Drs. Mellou and McManus, of Brownsville and Matamoras Medical Association, offered a resolution providing for a change in the Constitution and By-Laws so as to admit a physician to membership *in absentia*, upon the showing of the proper qualifications therein specified. Referred to committee on Constitution and By-Laws. Under the by-laws this must lie over a year.

Charges were preferred by Dr. J. J. Dial against Dr. M. K. Lott, of Belton, based on a pamphtet entitled "a Chapter in Medical Ethics as practiced by Dr. H. C. Ghent, by M. K, Lott," which pamphlet was said to have been distributed to the laity. Dr. Pitts moved to refer to Judicial Council, together with the pamphlet.

Dr. Lott rose to a point of personal privilege, and said he protested against being tried by twelve men from whose decision there is said to be no appeal.

The question as to the finality of the action of the Judicial Council came up, and the chair ruled that the decision of the Council is not final with reference to the last clause of first paragraph of article referring thereto—attention having been called to same by Dr. Talley. At request of President, V. P. Parsons took the chair.

Dr. Talley moved a substitute for Dr. Pitts motion, so as to have charges and specifications read in open session before being sent to Judicial Council. Lost.

Dr. Cummings asked if a member against whom charges had been preferred, desired it, could he not have the charges read in open session?

The chair decided in the negative. Dr. Talley appealed from the decision of the chair, and by a rising vote the chair was sustained.

The resignation of Dr. M. R. Brown, late of Galveston, now of Chicago, was read and accepted. Meeting adjourned.

AFTERNOON SESSION, SECOND DAY, APRIL 27.

The Association met at 3 o'clock, Vice-President Chilton in the chair.

The Section on Ophthalmology and Otology was called, Dr. T. J. Tyner, chairman, presiding. Dr. Tyner read a report reviewing the work of the year in that department of science. The paper was referred to the publishing committee with instructions to incorporate it in the minutes. Dr. Tyner was then excused to attend a sitting of the Judicial Council, and Dr. Chilton continued to occupy the chair

The first paper presented was upon Iritis, by Dr. Chilton. It was ably discussed by Dr. Saunders, who was specially invited to participate in the debate; by Drs. Nott, Pope, Leake, A. P. Brown, Jones, of Gonzales, Dudley and Chilton. It was referred to the publishing committee.

Dr. Becton was on motion excused from further attendance on the session, having a call home to attend an important case.

The second paper, "A Case of Neuro-Retinitis," by Dr. J. H. Smith, of Dallas, was about to be read, when a point of order was made that the section had already more than consumed its allotted time, two hours. The chair decided the point well taken. Several papers under this section were not reached, one by Dr. J. R. Briggs on the "Syringe in Ear Disease." All papers will be referred to the publishing committee, whether read or not.

The Section on Surgery was then called. Dr. Osborn, chairman of the section, announced that he had a report to make, but as it was lengthy and would consume a considerable amount of time, he would content himself by reading the captions of a number of cases. This he did, and the report was referred.

Under this section a paper entitled "A Case in Conservative Surgery" was read by Dr. J. D. Bass, and elicited a spirited discussion, which we are obliged to omit. It was discussed by Drs. Talley, Brown, Sears, Paine, of Comanche, and others.

Dr. J. R. Briggs sent up to the Secretary's desk a document addressed to the Secretary, with the written request to read it at once. The Secretary, Dr. Daniel, proceeded to read the following:

"*To the Judicial Council of the Texas State Medical Association, Greeting.*"

GENTLEMEN:

From certain facts herein specified, it becomes my duty as a member of the Texas State Medical Association, to present to you for your wise and just consideration, the following charges and specifications against one of our members, Dr. F. E. Daniel, of Austin.

1st. I hereby allege that Dr. F. E. Daniel did, contrary to the desire or request (by resolution or otherwise) of the Texas State Medical Association, and upon claims utterly groundless, assume for his journal (Daniel's Texas Medical Journal) the high prerogative of " the Official Organ of the Texas State Medical Association." I *protest* against such presumptious arrogance, and deny the right of any Journalist to unauthoritatively make such false and vain statements. I assert that outside of its "Transactions," the Texas State Medical Association *has no* recognized "*official organ*," and that all claims to the contrary are born of conceit and brought forth to gratify a vaulting ambition, to the detriment of the Association at large, and its members individually.

I further assert, that his false assumption, togethar with other efforts at self-aggrandizement, has reflected discredit upon the Association instead of upholding its principles and preserving harmony in our ranks.

Therefore, as a member of the Texas State Medical Association, I ask that Dr. F. E. Daniel be brought before your committee to answer to the false statement made and published by him, that his Journal was the "official organ" of the Association. I further ask, that the his liberty under the guise of such egotistical and assumed prerogative to criticise and cast personally, discredit and mortification on two of our ex-presidents be *questioned.*

2th. I desire to respectfully call your attention to page 28 in the "Transactions" for 1885 where the following language occurs: "The committee, by allowing five pages for advertising were enabled to save the Association $100, or 14 cents per copy in the price of printing the Transactions." On investigating the matter by writing to the treasurer, and obtaining information from other sources, I find as a *matter of foct* that the $100 referred to was not saved the Association; that the Association was not beneficiary of this $100, or *any part thereof*! I further assert that this $100 was appropriated by Dr. F. E. Daniel to his own personal benefit, which was contrary to his statement of the facts in the case, and also at variance, with business principles, honesty, and the confided trust in him reposed. I desire to say here, that for 1885 the Transactions was printed at Fort Worth, and was under the direct management of Dr. F. E. Daniel, and that this charge cannot in the least reflect upon our lamented Dr. Burt.

I believe that such a precedent of which Dr. F. E. Daniel has been guilty will, if not speedily reprimanded. lay the foundation for subsequent misappropriations of the funds belonging to our Association, and therefore ask that you justly and impartially consider this breach in all its relations, for the good of all concerned and to the injury (unjustly) of no one!

3rd I assert also without the possibility of successful contradiction, that for 1885 the printing of the "Transactions" only cost $400, *but*, that Dr. F. E. Daniel induced the printer—Mr. J. K. Millican—to send in the bill for $475; he (Dr. Daniel) appropriating the $75 *over charges* to *himself*, thereby bleeding the treasury a *second* time! That the "$100" gotten by Dr. Daniel for andvertising. and the $75 over charges, did not constitute his salary, is shown by the fact that at the last Houston meeting (1885) the publishing committee were paid $200 for their services, of which amount Dr. Daniel received $150. Thus summed up we find, that Dr. Daniel received, in *in three different ways* $325.00 for the year of 1885, and that the $100 and the $75 *really, and* in *fact, justly belonged* to the Association. While *it is a fact* that Dr. Daniel was only *one* of the publishing committee, nevertheless, it can be proven to your satisfactiou, that HE alone received and misappropriated the $175. If Dr. Daniel at the time, thought himself justifiable in keeping the $100, and the $75, for *his services*, he should at the next meeting in Houston, when the subject of paying the committee was up before the Association, have made the statement to that body, that he had *already* received for his services $175. But Dr. Daniel did not make any such statement or explanation, but on the contrary urged with his friends, that he be paid a second time, or receive additional sum, which was granted by the Association, *which body was ignorant* of the *fact* that he (Dr. Daniel) had been beneficiary of ever so small amount for his services.

Believing that such conduct is *dishonest*, and unbecoming one of our members, and that the precedent is a dangerous one, I submit these facts to your body, for a just and equitable consideration.

4th. (As a journalist Dr. Daniel has—under *false colors*—wrongfully, and *maliciously*, assumed the right to criticise the Association in its selection of its presidents; such criticism being engendered because he (Dr. Daniel) was not elected president at our last meeting; thereby manifesting a vindictive and bitter retaliation. It is not hard to see that any remark made by a journalist in our State derogatory to the high scientific, and honorable standing of our members, and officers, will throw discredit upon the Association as a scientific body, and lower us in the eyes of the scientific medical world at large).

5th (It can, in my *opinion*, be *fully sustained*, that Dr. Daniel is a "*fire brand*," a "*stirrer up of strife*" and a "*fomenter of discord*;" and that his retention in our body, can but create in the *future*, as in the *past*, discord and perpetual trouble in our ranks.

With the following explanation I close these charges, and desire the committee, or Judicial Council, to note the *fact*, that I have streniously avoided bringing any *personal* differences, existing between Dr. Daniel and myself, before your Council; *claiming as I think right* and *proper*, that the above greivances exist ALONE *between Dr. Daniel and the Texas State Medical Association*, I have no desire to annoy your Council with any *correspondence* between myself, Dr. Daniel, and others, as such "PRIVATE" affairs, should have NO place in your council, to take up your valuable time, and infringe upon your labors in behalf of the *fundamental interests of the Association.*

Having been informed, however, from reliable sources, that Dr. Daniel will in his defense, attempt to *recriminate* by showing you my *letters* to him and *others* (or get others to do so) I make the following statement in justice to myself:

I wrote very *severe* and *abusive* letters to Dr. Daniel and others, who were trying to *cover up his wrong acts;* my *sole* object being to observe, if by such *bitter letters* he (Dr. Daniel) could be made to make *plain*, and show his *undoubted* guilt. I was handsomely rewarded in my efforts, as was shortly afterward shown, by Dr. Daniel's rapid (and to others unexpected) visit to Fort Worth, to try and *patch* up and *smooth* over, his criminal acts. Upon receiving my bitter letters of accusations, Dr. Daniel went to the printer at Fort Worth at *once*, to try and get him, Mr. J. K. Millican, to give me a letter of retraction, which letter is in the hands of your chairman, Dr. H. M. Oliver. I claim I had a right to correspond in any way I thought proper, with Dr. Daniel or any one else (who was attempting to shield him) and that the detective method used by me was for the benefit of the Association, and could not possibly do me any good, *personally*. Having known for nearly a year, or at least *strongly suspicioned* that the funds of the Association had been misappropriated, In order to put the matter beyond doubt, I resorted to means, which would not under less aggrivating circumstances, have been proper and just. While I regard recrimination a poor method of ridding one's self of crime; *nevertheless*, if your council think (as will be charged against me) that I was not justifiable in using such *severe* means for the detection of this *crime*, I ask that you immediately *turn me out of your honorable and scientific body!* This I say, because I assure you that the letters I have written to Dr. Daniel and *others*, together with conversations I have had on the subject will be presented against me as evidence of my "malicious" (to Dr. Daniel) conduct. I ask that your council reject all such "private" correspondence to Dr. Daniel and *others*; but if you think it wise to entertain such an elaborate correspondence, I then ask the liberty of presenting about 200 pages of such letters written me by Dr. Daniel and others.

In conclusion I must say to you, that I expect to be accused of

"*persecution*," and that *other* matters may be rung in, in *lieu* of defensive evidence. I pray you to not allow me or any one else, among you with personal matters, but let the case stand upon the evidence which I shall point out to you in *person*, if allowed to so do by your council. I fully recognize the results of this step, I have taken in presenting such grave charges against one of our members, and fully know that the matter will be a *very disagreeable and unpleasant one*, but should I *shrink* from my duty, with such facts in my possession, I feel I would be as guilty as Dr. Daniel, were I not to present them. Thus gentlemen I close this explanation, and put myself in your hands, together with these papers, and ask that you be as merciful as the *demands* of the *case*, and *justice* will *tolerate!* If at any time during your sessions, my presence may be found necessary, command me.

Faithfully and fraternally yours,

J. R. BRIGGS, M. D.

Dr. Daniel rose to a point of personal privilege and asked to be permitted to speak in his defense, a few words. The Chairman, Vice President Chilton, ruled him out of order. A motion was made by Dr. Tyner that Dr. Daniel be permitted to reply. Ruled out of order. The matter was pressed by several members, and finally Dr. Briggs moved that Dr. Daniel be permitted to reply to the charges. Permission being obtained, Dr. Daniel said :

"I deny any criminality in the matter charged, and repudiate it in toto. I am prepared to prove the entire allegations in every particular, as false as hell, and to vindicate my character, which for twenty-five years as a physician, no man has ever dared to assail ! I am not only prepared to disprove the charges in every particular, but also to show that it is *not* a conscientious charge brought by one brother against another, solely for "the protection of the profession," as asserted,—but a *malicious*, *vindictive*, *bloodthirsty* demand for *revenge!!* for an assumed injury ; for the gentleman says that he has been in possession of the "information" a year. In that time he has overwhelmed me with "friendly" letters—pledging life-long friendship and lasting gratitude for services therein acknowledged—and all signed "your true friend"—yet, not until a short time ago, when he assumed that I had aided Wallace and others, in criticising his "prize essay"—did his "conscience" suggest that it was his "duty," to bring such charges. I demand a thorough investigation into all my official acts."

The document was referred to the Judicial Council. The Judicial Council next day reported as follows :

"We, the Judicial Council of the Texas State Medical Association of the 19th session, held at Austin, April, 1887, after full investigation of the charges against F. E. Daniel, M. D., do exhonorate him from any criminality, or intentional criminality, in the alleged misappropriation of any funds in his hands. In regard to the funds realized from the advertisements published in the Transactions, and appropriated to his own use, we believe, he acted on what he conceived to be good and sufficient reasons. Furthermore, we exhonorate him from all other charges brought against him."

[Signed], M. H. OLIVER, Pres.
J. W. GARNETT, Sec.

Dr. Daniel thanked the Association for this act of simple justice, and taking occasion to refer to one point of complaint, said :

" With reference to the editorial in last July's issue of the JOURNAL, "Misguided Members and the President's Address," I desire to say that I have disclaimed through the JOURNAL, and I here now on this floor, disclaim any intentions to reflect upon the personal, or professional character of either Drs. Bector or Brown, or to injure them. My language and my animus were both misunderstood; and whatever construction it may have been thought susceptible of, it was far from my intention to cast aspersions on them. I attributed to them simply an error of judgmeut in referring to the subject of religion and evolution in their addresses, and thought it was not only out of place, but calculated to engender strife. When a member, three years ago, introduced the subject, I foresaw an element of discord ; when a year later, Dr. Brown referred to it and replied to certain remarks, it seemed the Association was drifting on to dangerous ground, and, when still later, Dr. Becton, in his presidential address, referred to certain members, who held adverse views to his own as "misguided," I thought it was time for the JOURNAL, which was established for the declared purpose of promoting organization of the Medical profession of Texas, and its advancement and elevation, to sound a warning. Such was conscientiously the motive of the editorial,—to warn the Association of the danger of continuing the discussion of a subject which all must recognize as foreign to the objects and dangerous to the peace and harmony of the body ; and, while I make this explanation, in justice to myself and to the distinguished gentlemen who feel aggrieved at my remarks, I have nothing to retract as to the subject matter of the article, i. e., that "the majority of the profession deprecate the introduction and discussion of a religious question in the Association meetings ;" and that no one is authorized to speak as to the religious sentiments of the body. I am much pleased to state that after a conversation with Dr. Becton in which the above, in substance was said, Dr. Becton expressed himself satisfied, and assured me of a complete restoration of friendly relations between us,—of which I don't hesitate to say I feel proud : and, having done all that a gentleman could be expected to do, or say, in the premises,

I only wish that I was able to say the same with regard to Dr. Brown, that he is again my friend. (Dr. Brown and Dr. Becton were both absent.)

Pending motion to adjourn, Dr. Clark moved that Dr. Briggs be expelled. Motion seconded by several. Ruled out of order, and motion to adjourn prevailed.

THURSDAY, APRIL 28—MORNING SESSION.

Meeting called to order by President Nott. Minutes of yesterday read and approved, with corrections.

The Committee on Collective Investigation of Disease was called, and chairman Sam R. Burroughs read his report (omitted for want of space), showing the kind and amount of work done in that field, and the expenditures incident thereto.

On motion of Dr. Sears, the report was adopted, and the committee discharged.

Dr. Tally, delegate to Foreign Medical Association, made an interesting verbal report. contrasting our own methods with those of other societies.

Dr. Cupples, chairman of the Committee on Legislation, submitted the following

REPORT OF COMMITTEE ON LEGISLATION.

*Dr. T. H. Nott, M. D., President Texas State Medical Association:*

SIR.—Your Committee on Legislation have the honor to report, and that with great regret, that no measure has been devised which, while beneficial to our people, could present any hope of acceptance by a singularly impracticable and unpractical legislature. In consequence, it was deemed more prudent, in view of action in the future, to avoid defeat, by inaction, than to court it by any appeal to the Legislature in its then temper. And in this connection, your committee would respectfully suggest that naked reason will never secure from a legislative body the recognition due to the medical profession in the interest of the public health, and that the pressure of public opinion must be invoked to reach minds otherwise inaccessible.

For this end, your committee proposes that an attempt, contiuuous, not intermitting, be made to enlist every physician in our State as an advocate of a law to organize boards of health, and of kindred measures—and that each one of these physicians, in his own circle of friends and patients, enlighten the people on the paramount questions of vital statistics and preventive or State medicine.

Respectfully submitted. CUPPLES, Chairman.

The Nominating Committee was organized, and elected Dr. J. B. Robertson chairman. On motion, the meeting was postponed till the report of the Judicial council had been made.

Dr. Sears moved that in future advertisements be excluded from the pages of the transactions, and that the Publishing Committee be paid $300 out of the Association Treasury for time and labor in the compilation. Carried.

The report of the chairman of Section on Obstetrics, Dr. J. W. McLaughlin, was, by request, read by title and referred.

The report of the chairman of Section on Electro Therapeutics, Dr. F. T. Paine, was read—"Three Years Work with Electricity in the Treatment of Disease." Referred.

A handsome volume of Dr. Jos. Jones' Memoirs was presented by the author, through Dr. Q. C. Smith. Accepted with thanks, and Secretary instructed to acknowledge receipt.

Report of chairman on Section on Dermatology, Dr. H. L. Taylor, was called for. Dr. Taylor made a verbal report, and promised to reduce it to writing in time for the Transactions.

Dr. Dudley moved to change the sections so as to throw venereal diseases under the head of Dermatology; and Med. Botany under head of Chemistry. Laid on table.

Adjourned till 3 p. m.

THURSDAY, APRIL 28—AFTERNOON SESSION.

Meeting called to order. President Nott in the chair.

The President handed the Secretary the following charges by Dr. J. J. Dial against Dr. J. R. Briggs—which, by order, was read and referred to Judicial Council.

*To The Judicial Council:*

GENTLEMEN.—I herein present charges against Dr. J. R. Briggs, for his action in preferring charges against Dr. F. E. Daniel, and ask that he be either expelled or exhonorated from your body at once. I do this both in justice to the Association and Dr. Briggs and his friends. Respectfully,

J. J. DIAL.

Dr. Daniel declined to appear or give evidence against him, and the Judicial Council reported as follows:

The Judicial Council of the the Texas State Medical Association having investigated the charges against Dr. J. R. Briggs, respectfully submit: That we do not deem it a crime for one member to prefer charges against another in this Association; and, as we can find no other specification in the charge, hereby recommend his exhonoration.

M. H. OLIVER, President,
J. W. GARNETT, Secr'y.

On the charges vs. M. K. Lott, the Judicial Council reported as follows:

Charges and specifications, after being investigated, were sustained, and the Council recommend such action as the Association may deem proper.

M. H. OLIVER, President,
J. W. GARNETT, Sec'y.

Dr. Lott denounced the charges as false, and said he had taken every legitimate means of settling the difficulty between Dr. Ghent and himself, and the profession at Belton would not settle it, and that he had appealed to the only recourse left him.

A motion to expell Dr. Lott, by Dr. C. F. Paine, was lost, 82 yeas and 42 nays (tally kept by the Journal clerk of the Legislature). A motion to repremand was lost, and a motion to suspend for one year prevailed unanimously.

Section on Obstetrics was called. Dr. McLaughlin being excused, Dr. Fred Terrell, Secretary, occupied the chair.

Dr. J. D. Osborn read a paper on "Puerperal Eclampsia treated Hypodermatically."

The paper was discussed at considerable length and elicited much interest, and developed several modes of treatment as practiced by members. Dr. O. L. Williams had not seen a case which did not yield to timely and sufficient blood-letting, while others condemn that method and pinned their faith to veratrum. Dr. Sears had faith in saturated solution of Epsom salts in hot water, after quieting patient with chloroform. Dr. Tally believed in building up the patient in anticipation, by tonics and nutritous diet, and argued that the convulsion was a result of a dyscrasia, or debility. D. Enochs said all his patients die in spite of treatment. Dr. Osborn said he could diagnose Eclampsia by the presence of albuminuria. Dr. Bowers gives 5 to 10 gtt: doses of verat viride ter die. Dr. Osborne's paper referred with instructions.

Dr. H. A. West read a valuable paper on "Observations on two cases Placenta Prævia." Referred after discussion with special i nstructions.

Dr. Bowers read a paper,"Complicated Cases of Labor." Referred.

A number of other papers under this section were read by title and referred.

Section on Gynæcology called. Dr. F. H. Tucker, Chairman, presiding.

Dr. O. Eastland read for Dr. B. E. Hadra, a paper on "Tracheolorrhaphy, and some Suggestions for its Modification." Discussed and referred.

A paper by Dr. Pennington on "Removal of Uterine Polypi" was read and referred.

Section on Surgery recalled by permission. Dr. J. C. Jones read a paper giving an account of a Laparotomy for Intestinal Obstruction, etc.; referred with instructions.

Papers were read by Drs. J. P. Oliver, E. L. Ward and L. B. Creath, and referred. Dr. Tucker read a paper on Adherent Prepuce and exhibited a specimen of monstrosity.

Dr. T. D. Wooten delivered an address before the Association Thursday night in the University. Dr. J. F. Y. Paine moved a vote of thanks, and that the address be published in the Transactions. Carried.

Dr. R. P. Tally preferred charges against Dr. H. C. Ghent, for conduct unbecoming a gentleman and a member, and specified certain letters which the mover characterized as "infamous and libelous," and that they were for the purpose of securing the presidency. Referred to a special Judicial Council, composed of Drs. R. G. Williams, P. J. Bowers, J. C. J. King, F. M. Pitts, J. F. Y. Paine, A. M. Douglass, T. J. Tyner, H. K. Leake, J. W. McLaughlin, O. I. Halbert, J. L. Carter, W. A. Duringer,—the original Council having adjourned and same gone home.

After examination the Judicial Council reported as follows: "We find the charges and specifications against H. C. Ghent, M. D., flimsy and puerile and entirely unsustained by the evidence produced, and absolutely unworthy of consideration. We exhonorate Dr. Ghent of even a suspicion of guilt."

J. W. McLaughlin, Pres.
T. J. Tyner, Sec.

On motion the committee on prize essays was discharged. Adjourned.

Thursday night at 8 o'clock, Dr. Nott, the retiring president, delivered his address to a large and select audience of ladies and gentlemen, together with a fair attendance of members. We regret, that we cannot publish the address in this issue. Dr. Wooten's address followed.

FRIDAY, APRIL 29. MORNING SESSION.

Meeting called to order. President Nott in the chair. Secretary absent temporarily: Dr. Osborn acted for him. On motion, reading minutes was dispensed with.

Dr. Osborn, of Committee on President's recommendations reported, advising the adoption of the suggestions submitted.

Dr. Talley read his report as chairman of Committee on Constitution and By-Laws appointed at Dallas (Report not in Secretary's hands). Dr. Osborn moved that the committee be continued, and that the report be published in the next volume of Transactions, and made the special order for first day of next meeting. Adopted.

The nominating committee reported the following

OFFICERS FOR THE ENSUING YEAR:

President, Sam. R. Burroughs, of Leon county.
First Vice-President, R. T. Knox, of Gonzales.
Second Vice-President, A. M. Douglass, of Hill county.
Third Yice-President, A. A. Terhune, of Jefferson.
Secretary, F. E. Daniel, of Austin. (5 years).
Treasurer, J. Larendon, of Houston, (5 years).
Judicial Council: P. C. Coleman, R. Rutherford, M. Knox, R. C. Nettles. (New members).
Section on Practice, C. M. Ramsdell, Chairman.
Section on Obstetrics, J. J. Dial, Chairman.
Section on Surgery and Anatomy, A. W. Fly, Chairman.
Section on Med. Jurisprudence, etc., H. A. West, Chairman.
Section on State Medicine, R. M. Swearingen, Chairman.
Section on Gynæcology, G. W. Christian, Chairman.
Section on Ophthalmology, B. A. Pope, Chairman.
Section on Dermatology, H. W, Dudley,, Chairman.
Section on Electro Therapeutics, R. W. Knox, Chairman.

COMMITTEE ON PUBLICATION.

F. E. Daniel, Chairman; R. M. Swearingen, J. W. McLaughlin.

Committee Collection Surgical Cases: Geo. Cupples, Chairman. E. J. Beall, J. M. Pace, B. F. Eades, D. F. Stuart, A. G. Clopton.

Delegates to Associations—omitted till next issue.

Committee of Arrangements: J. F. Y. Paine, chm., to select committee. Place of meeting, Galveston, 4th Tuesday in April, '88.

Committee on Prize Essays—omitted till next issue.

Committee on Revision Constitution and By-Laws—omitted till next issue.

By resolution of J. R. Johnson, the Publishing Committee is authorized to reject any papers that in their judgment should not be published in the Transactions, independent of previous instructions. Adjourned.

During the Session 52 new members were admitted, and several new county associations —names of all of whom and which will be given in next issue.

Dr. Florence E. Collins, of Austin, Secretary of Travis County Medical Association, was elected a member by a rising vote·

The Opera of Balshazzar, given by the Austin Musical Union, (amateurs) under contract with the committee of arrangements for the especial entertainment of the delegates, was largely attended and much enjoyed. The several receptions. and the entertainments at the University and other places were also much enjoyed; and delegates returned home much pleased with the nineteenth annual convention of the T. S. M. A., and their trip to the Capital.

