# Errata

**Published:** 1887-06

**Authors:** 


ERRATA.—In the Synopsis of Proceedings Texas State Medical Association, published in our last number (May) the "intelligent compositor" read "June" for "April," and made us date the meeting a month ahead of that issue ! Also, in the matter of Briggs vs. Daniel, in writing from memory, we inadvertently used the word "alleged." It does not occur in the report of the Judicial Council, we see by reference to the original.

